# Supplementary material for: Combination of Biochemical, Molecular, and Synchrotron-Radiation-Based Techniques to Study the Effects of Silicon in Tomato (Solanum Lycopersicum L.)
Source: Int J Mol Sci. 2022 Dec 13;23(24):15837. doi: 10.3390/ijms232415837 (PMC9785873; doi:10.3390/ijms232415837)
Supplement: Supplementary file 1 [file ijms-23-15837-s001.zip › ijms-2007121-supplementary.pdf]

Table S1. Literature on the state of the art of Silicon capacity to alleviate abiotic stress in tomato.

Stress type	Plant part	Si treatment concentration	Treatment time	Anti-ROS enzymes analysis	Anti-ROS Non-enzymes analysis	Genes analysed	Reference
Salt stress 100 mM NaCl	leaf	2.5 mM soil	27 days	SOD, APx, CAT, G-POD	Chl content, MDA, H_2O_2 content		[104]
Drought polyethylene glycol (PEG, 6000)	Leaf roots	1.5 mM hydroponics	21 days		Sulfate and ammonium Metabolomics	RTqPCR: SlJUB1, SlASS, SlASL, SlMS, SlGAD1, SlGAD3, SlP5CR, SlP5CS, SlGR, SlACTIN, SlEF1α, SlCAC, SlSAND	[48]
Drought 1.0% PEG	roots	0.6 mM Si pretreatment 1.2 mM Si hydroponics	3, 5, 12 days	SOD, POD, CAT	TEM, O_2^- productivity rate, H_2O_2 content, MDA, content of soluble sugar, soluble protein, proline		[105]
Drought 1.0% PEG	leaf	0.6 mM Si pretreatment 1.2 mMSi hydroponics	3,5,8,12 days	proteomic analysis	Gas exchange, SEM, O_2^- productivity rate, H_2O_2 content, MDA		[106]
Ralstonia solanacearum	leaf roots	1.4 mM $Si(OH)_4$	72 h post inoculation			phosphoglycerate kinase genes (PGK), a-tubulin (TUB) and actin (ACT)	[107]
Ralstonia solanacearum	leaf	1.4 mM $Si(OH)_4$	72 h post inoculation			microarray e qRTPCR: WRKY-IId5, JERF3 TSRF1, ACCO, PR1, FD-I, GLU, CHI-II, POD, PAL, AGP-PGIP, PGK	[108]
Sodic soils NaCl B	shoot root	2.5 mM and 5.0 mM Si soil	4 months	LOX, SOD, CAT, APX	stomatal resistance (SR), MDA, membrane permeability (MP), proline, H_2O_2 content, non-enzymatic total antioxidant activity		[109]
Pythium aphanidermatum	roots	1.4 mM silicic acid	2 days		double-sandwich ELISA		[110]
Fusarium oxysporum	roots crowns stems	100 mg Si/l. in sand	4 weeks after inoculation		Dry weight, Si content, disease assessment		[111]
High pH stress	leaf roots	1 mM Si 100 µM SA Soil Si, SA, Si+SA	5 weeks	APX, CAT, POD, PPO (polyphenoloxidase)	Chl*a*, Chl*b*, leaf relative water content (LRWC) lipid peroxidation, • O_2^- productivity rate; Si quantification, SA quantification, ABA quantification	RTqPCR: LSi1, *LHA1, LHA2, ICS, SAMT1, SABP2, SAMT, SABP2, ICS, PAL1, PAL2, CAT, POD, APX, SOD*	[112]
Heat stress (43 ± 0.5 °C).	roots shoots leaf	50 mL of 1mM Si as Na_2SiO_3	10 days	total proteins, POD, CAT, PPO, APX	Photosynthetic pigments, relative water content (LRWC), Silicon analysis,	qRT-PCR: CAT, APX POD GR Cu/ZnSOD GST NCED1 ICS PAL PR1b1 PR-P2 HsfA1a HsfA2, SlHsfA3, HsfA7,	[113]

					lipid peroxidation, SA quantification, ABA quantification	HsfA1b,DREB2, MAPK1	
Salt stress 150 mM NaCl	root shoot leaf	2 mM Si sand	15 days	SOD, CAT	Dry weights of plant tissues, leaf area, pigment concentration, photosynthetic parameters (photosynthetic rate, transpiration rate, stomatal conductance, intercellular CO_2 concentration); leaf relative water content (RWC), root morphology traits, root hydraulic conductance, root osmotic potential, membrane electrolyte leakage, MDA, H_2O_2 contents, contents of non-protein thiols and ascorbic acid		[114]
Salt stress 25 mM 50 mM	roots	2.5 mM Si hydroponics	5 days	proteomic analysis SOD	silicon content, lipid peroxidation, histochemical localizations of H_2O_2 in roots	RTqPCR: salt stress responses (leDREB-1, leDREB-2, leDREB-3), antioxidants (leAPX, leSOD, leCAT) and Si transport (leLsi-1, leLsi-2 leLsi-3)	[115]
Salt stress 25 mM 50 mM	chloropla st	2.5 mM Si (Na_2SiO_3) hydroponics	14 days	proteomic analysis	Lipid peroxidation, H_2O_2 localization, visualization of O_2^-, total chlorophyll and carotenoid contents, photosynthetic rate, stomatal conductance and transpiration levels		[116]
Salinity stress 80mM NaCl	roots leaf	2.5 mM Si hydroponics	30 days		ion analysis Na^+ and K^+ Cl^-, Si, water uptake, leaf water potential, stomatal conductance to water vapour (gs), net CO_2 assimilation (ACO_2), transpiration (E) rates, plant water content, water use efficiency		[117]
Drought 10% (w/v) PEG-6000	root leaves	2.5 mM Si hydroponics	7 days	SOD, CAT	Photosynthetic gas exchange, relative water content, root hydraulic conductance, root osmotic potential, proline, relative electrolyte leakage of roots, MDA, ROS levels, GSH, AsA	*qPCR: SlPIP1;3 SlPIP1; 5SlPIP2;6*	[118]
Cadmium pollution	roots leaves	2 mM $Na_2SiO_3{\cdot}9H_2O$	2 weeks pretreatment	CAT, SOD, APX, GR	Cd and Si concentration, organic acid contents,		[119]

100 µM $CdCl_2$		pretreatment and with Cd	15 days treatment		root cell wall isolation and analysis (FTIR), PTS apoplastic transport of Cd, lipid peroxidation, H_2O_2 content, membrane stability index (MSI), GSH, AsA		
Low P<br>P 0.44 mM	root<br>leaf	1.5 Mm Si	3 weeks	SOD, POD, CAT	Biomass, root morphology, Chl, Gas exchange parameters, water use effciency (WUE), lipid peroxidation, H_2O_2 content, superoxide (O_2), osmotic potential, soluble sugar, soluble protein proline, free amino acid and organic acid contents, Si content, concentrations of K, Na, Ca, Mg, Fe, Mn, Zn, and Cu	Transcriptomics of phospholipids	[73]

Table S2. Morphological analysis of the aerial part, roots and fruits of tomato plants cvs Aragon and Gladis.

		AERIAL PART				ROOTS				FRUITS			
		**lenght (cm)**		**weight (g)**		**lenght (cm)**		**weight (g)**		**number**		**weight (g)**	
		**mean**	**sd**	**mean**	**sd**	**mean**	**sd**	**mean**	**sd**	**mean**	**sd**	**mean**	**sd**
**ARAGON**	**NT**	108,0	14,1	229,0	43,8	47,5	0,7	50,5	9,2	18,0	11,3	260,0	175,4
	**Low**	110,0	7,1	208,0	19,8	51,0	0,0	42,0	4,2	14,5	0,7	275,5	43,1
	**High**	100,5	19,1	179,5	41,7	45,5	7,8	35,5	2,1	13,5	2,1	309,5	13,4
**GLADIS**	**NT**	108,5	2,1	255,5	81,3	44,0	2,8	51,0	36,8	18,0	5,6	271,0	100,4
	**Low**	112,0	2,8	210,5	0,7	49,0	2,8	57,0	9,9	17,5	4,9	268,5	65,8
	**High**	107,5	10,6	239,0	15,6	46,5	0,7	53,5	0,7	15,5	6,4	94,0	94,8

Table S3. List of the primer used for gene expression analysis by qRT-PCR.

ID TARGET GENES	FW	RV	ENCODING PROTEIN	Reference
COX1	TATCTAGGCATGGTTTATGCC	CTGCTAAGGGCATTCCATCC	cytochrome c oxidase subunit 1	[120]
GAPDH	ACCACAAATTGCCTTGCTCCCTTG	ATCAACGGTCTTCTGAGTGGCTGT	Glyceraldehyde-3-phosphate dehydrogenase B-tubulina	[121]
CAO	AACCACGAAGAACGCTGAAT	TTGCTCAAAGCAATCAATCG	Chlorophyllide a oxygenase, chloroplastic	[122]
SlHXK3	TAATGATGGTTCAGGCGTTG	CAGGCACTTTTGGTTGTGTC	Hexokinases 3	[123]
CNGC2	TCGCAAGTATGCTGCAATGTT	GTCGAAAAATATGCCAAACGAGG	Cyclic nucleotide-gated ion channel 2	[124]
Solyc06g036100	GGCAGGGAACCTTTCACTCT	TAGCCAAAGTGTTGAGCCCG	Putative LSI2-like silicon	[125]

			efflux transporter
ACTIN	ACCCTCCAATCCAGACACTG	TGACAGGATGAGCAAGGAAA	Actin

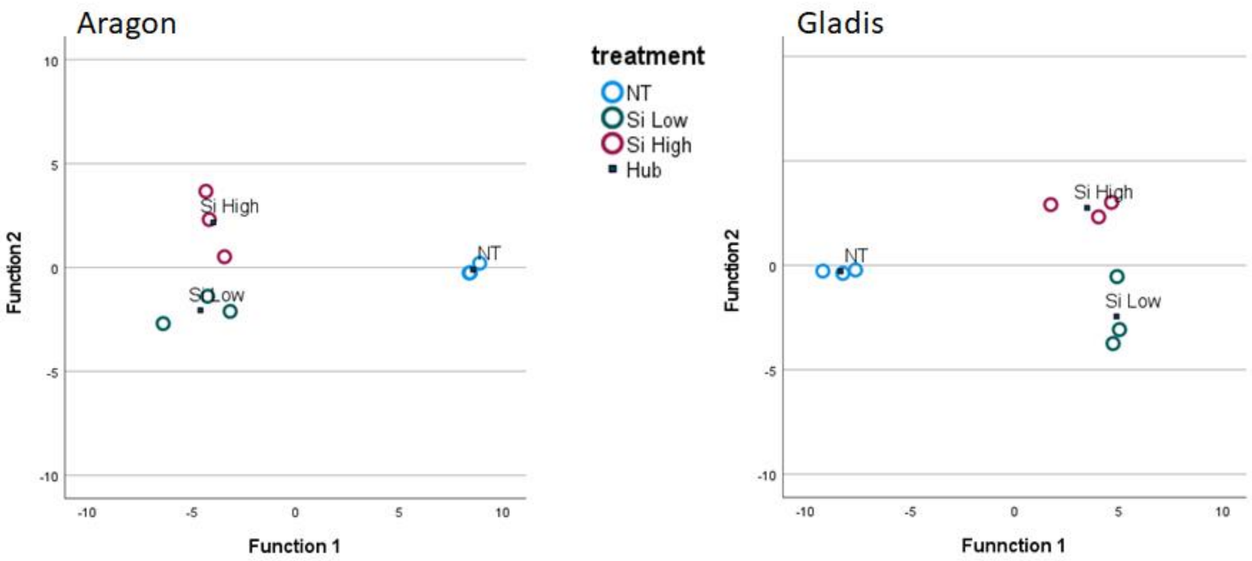

Figure S1. Discriminant Canonical Function analysis of Morphological Analyses. Blue=Control, Green= Low Si, Red= High Si. Blue square= hub of the group.

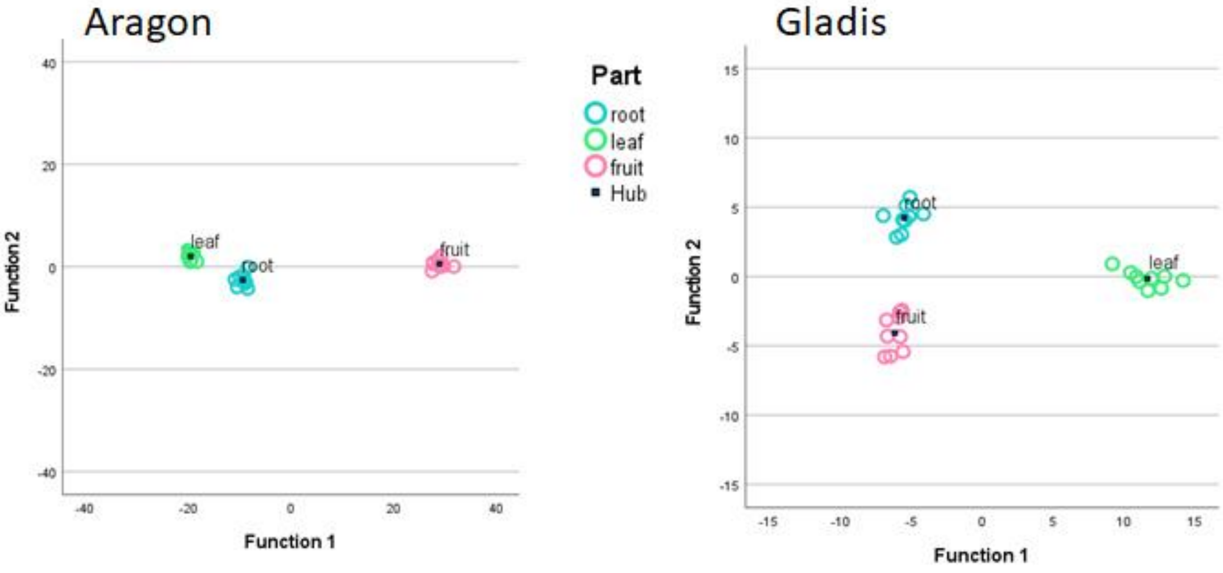

Figure S2. Discriminant Canonical Function Analysis of Elements per parts. Blue=leaf, Green= root, Red= fruit. Blue square= hub of the group.

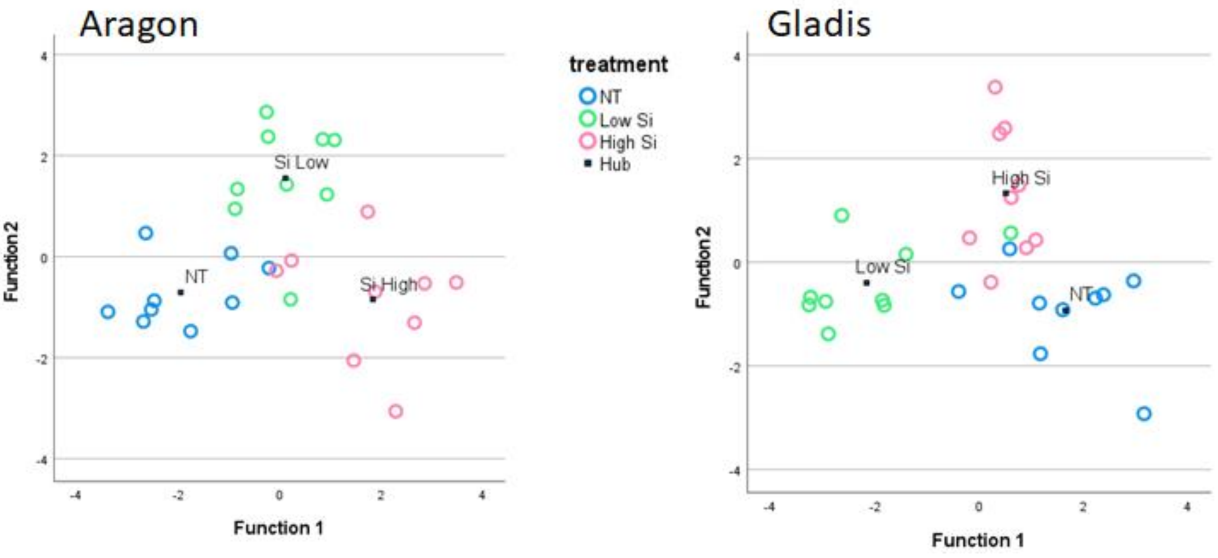

Figure S3. Discriminant Canonical Function analysis of Elements per treatments. Blue=Control, Green= Low Si, Red= High Si. Blue square= hub of the group.

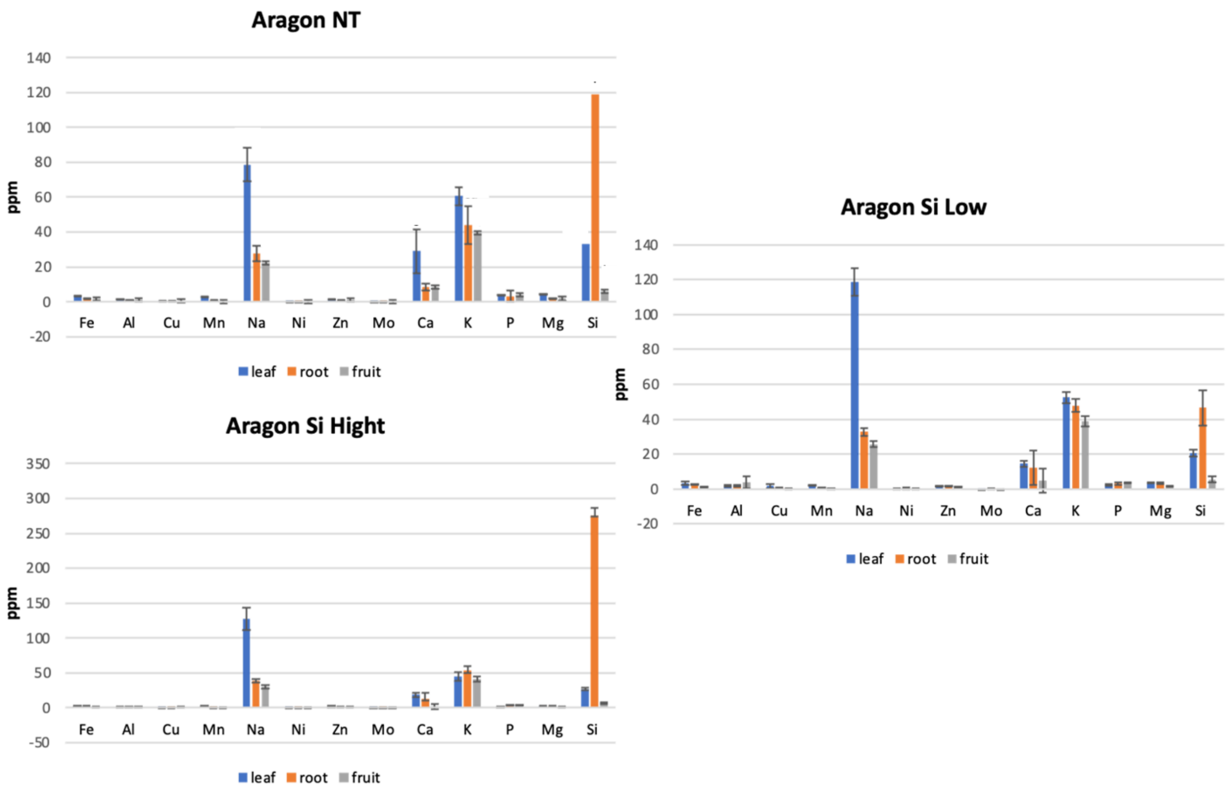

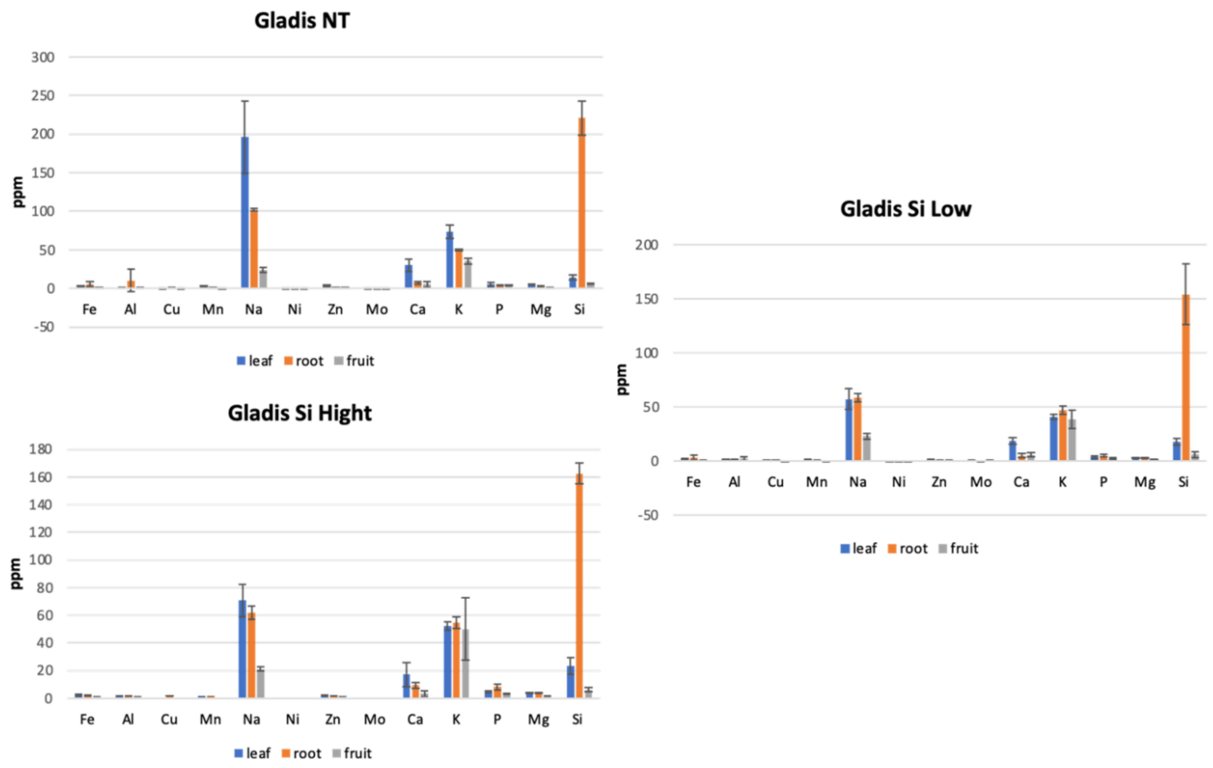

Figure S4. a: Chemical analyses of the elements in the various organs according to treatment for the cultivar Aragon. ANOVA has been performed followed by post hoc Tukey's HSD test with p value P<0.005. The P values for Tukey's Multiple Comparisons for each element are in Tables S11. **b:** Chemical analyses of the elements in the various organs according to treatment for the cultivar Gladis ANOVA has been performed followed by post hoc Tukey's HSD test with p value P<0.005. The P values for Tukey's Multiple Comparisons for each element are in Tables S12.

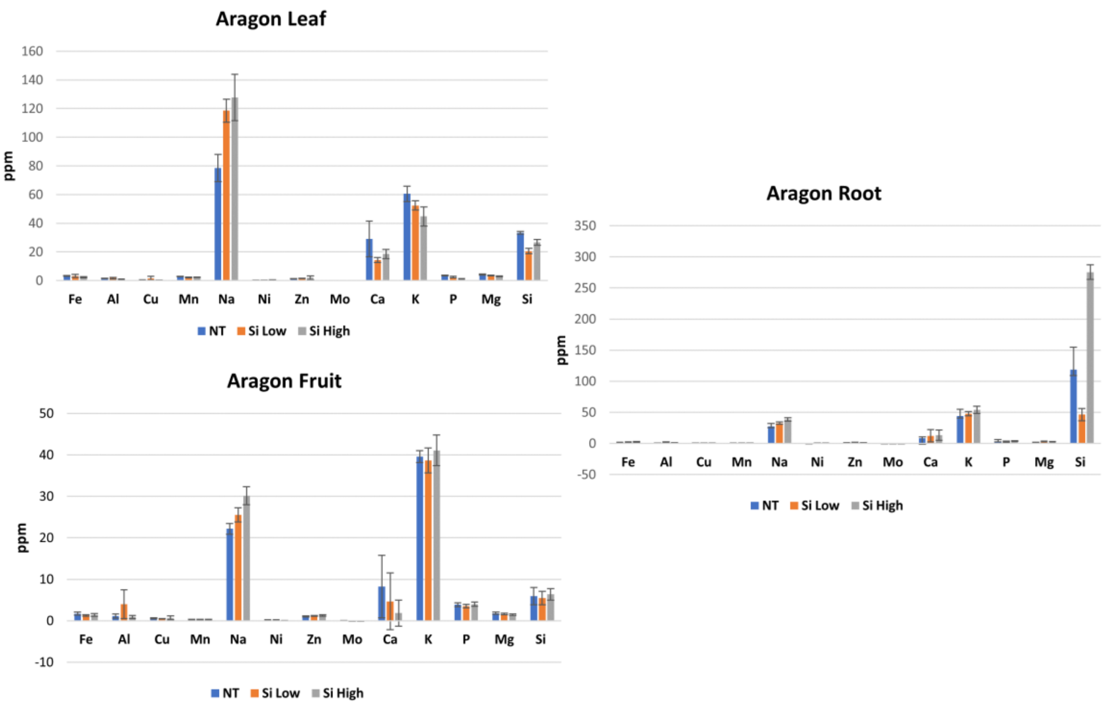

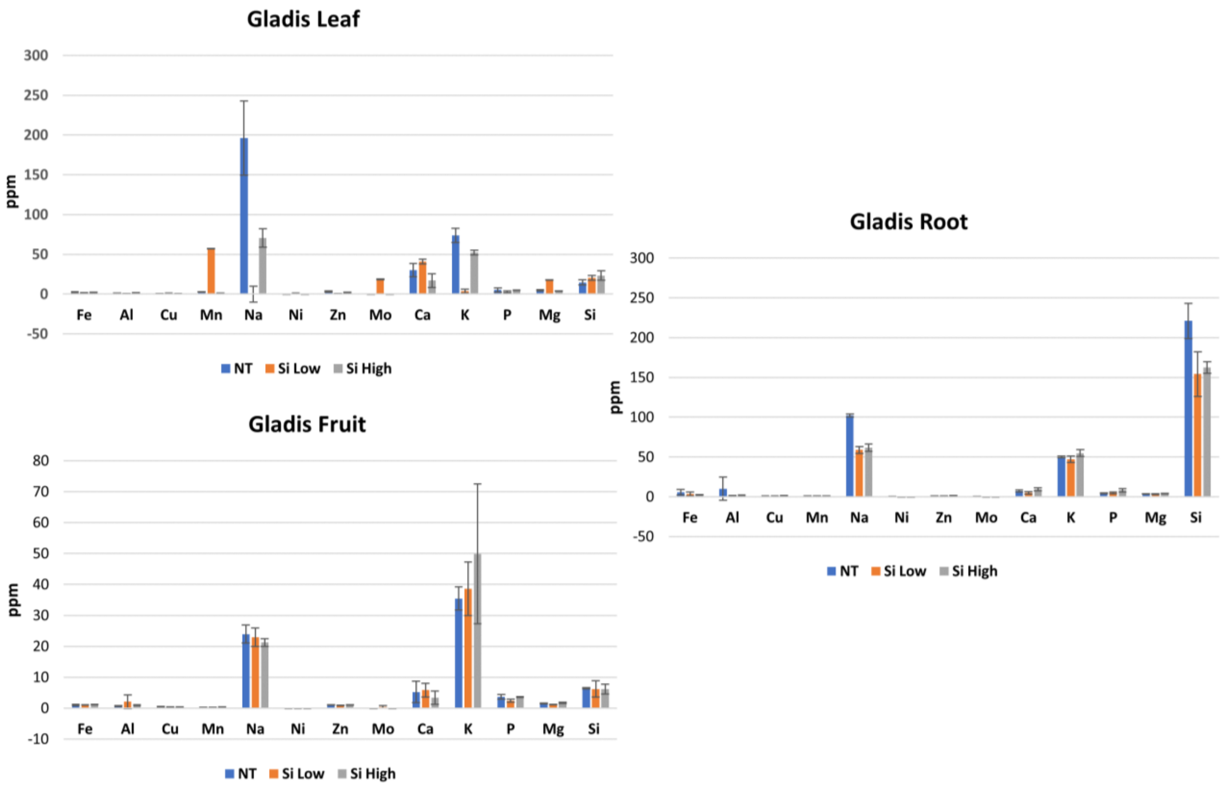

Figure S5. a: Chemical analyses of the elements in the various organs according to plant parts for the cultivar Aragon. ANOVA has been performed followed by post hoc Tukey's HSD test with p value P<0.005. The P values for Tukey's Multiple Comparisons for each element are in Tables S10. **b:** Chemical analyses of the elements in the various organs according to plant parts for the cultivar Gladis. ANOVA has been performed followed by post hoc Tukey's HSD test with p value P<0.005. The P values for Tukey's Multiple Comparisons for each element are in Tables S7.

Table S4. Aragon, Tukey HSD Multiple comparison for treatment. NT= not treated; Si Low= low Si treatment; Si High= High Si treatment.

**TP**		**NT**	**Si Low**	**Si High**	**MDA**		**NT**	**Si Low**	**Si High**
	**NT**		0,910572	0,987746		**NT**		0,482255	0,599385
	**Si Low**	0,910572		0,962505		**Si Low**	0,482255		0,979357
	**Si High**	0,987746	0,962505			**Si High**	0,599385	0,979357	
**SOD**		**NT**	**Si Low**	**Si High**	**POD**		**NT**	**Si Low**	**Si High**
	**NT**		0,950136	0,673846		**NT**		0,004615	0,022338
	**Si Low**	0,950136		0,488496		**Si Low**	0,004615		0,782454
	**Si High**	0,673846	0,488496			**Si High**	0,022338	0,782454	
**GST**		**NT**	**Si Low**	**Si High**	**GPX**		**NT**	**Si Low**	**Si High**
	**NT**		0,348951	0,500448		**NT**		0,628526	0,697908
	**Si Low**	0,348951		0,958601		**Si Low**	0,628526		0,992961
	**Si High**	0,500448	0,958601			**Si High**	0,697908	0,992961	
**PRO**		**NT**	**Si Low**	**Si High**	**H2O2**		**NT**	**Si Low**	**Si High**
	**NT**		0,054497	0,317964		**NT**		0,165002	0,090197
	**Si Low**	0,054497		0,598575		**Si Low**	0,165002		0,944077
	**Si High**	0,317964	0,598575			**Si High**	0,090197	0,944077	
**CAT**		**NT**	**Si Low**	**Si High**	**APX**		**NT**	**Si Low**	**Si High**
	**NT**		0,90723	0,136602		**NT**		0,581703	0,987861
	**Si Low**	0,90723		0,278558		**Si Low**	0,581703		0,492142
	**Si High**	0,136602	0,278558			**Si High**	0,987861	0,492142	
**LOX**		**NT**	**Si Low**	**Si High**					
	**NT**		0,878635	0,839059					
	**Si Low**	0,878635		0,996429					

	Si High	0,839059	0,996429	

Table S5. Aragon, Tukey HSD Multiple Comparisons Aragon Parts. L= leaf; R= root; F= fruit.

		NT	Si Low	Si High			NT	Si Low	Si High
TP	NT		0,997156	0,999676	MDA	NT		0,98941	0,999696
	Si Low	0,997156		0,99875		Si Low	0,98941		0,992671
	Si High	0,999676	0,99875			Si High	0,999696	0,992671	
		NT	Si Low	Si High			NT	Si Low	Si High
SOD	NT		0,039657	0,360336	POD	NT		0,988077	0,420481
	Si Low	0,039657		0,001479		Si Low	0,988077		0,504759
	Si High	0,360336	0,001479			Si High	0,420481	0,504759	
		NT	Si Low	Si High			NT	Si Low	Si High
GST	NT		0,828361	0,458865	GPX	NT		0,831244	0,468864
	Si Low	0,828361		0,808952		Si Low	0,831244		0,199575
	Si High	0,458865	0,808952			Si High	0,468864	0,199575	
		NT	Si Low	Si High			NT	Si Low	Si High
Pro	NT		0,845464	0,75692	H2O2	NT		0,653481	0,791069
	Si Low	0,845464		0,4257		Si Low	0,653481		0,970913
	Si High	0,75692	0,4257			Si High	0,791069	0,970913	
		NT	Si Low	Si High			NT	Si Low	Si High
CAT	NT		0,419719	0,464662	APX	NT		0,924684	0,987084
	Si Low	0,419719		0,051846		Si Low	0,924684		0,972765
	Si High	0,464662	0,051846			Si High	0,987084	0,972765	
		NT	Si Low	Si High					
LOX	NT		0,07603	0,015622					
	Si Low	0,07603		0,748747					
	Si High	0,015622	0,748747						

Table S6. Gladis, Tukey HSD Multiple Comparison for treatment. NT= not treated; Si Low= low Si treatment; Si High= High Si treatment.

		NT	Si Low	Si High			NT	Si Low	Si High
TP	NT		0,910572	0,987746	MDA	NT		0,482255	0,599385
	Si Low	0,910572		0,962505		Si Low	0,482255		0,979357
	Si High	0,987746	0,962505			Si High	0,599385	0,979357	
		NT	Si Low	Si High			NT	Si Low	Si High
SOD	NT		0,950136	0,673846	POD	NT		0,004615	0,022338
	Si Low	0,950136		0,488496		Si Low	0,004615		0,782454
	Si High	0,673846	0,488496			Si High	0,022338	0,782454	
		NT	Si Low	Si High			NT	Si Low	Si High
GST	NT		0,348951	0,500448	GPX	NT		0,628526	0,697908
	Si Low	0,348951		0,958601		Si Low	0,628526		0,992961
	Si High	0,500448	0,958601			Si High	0,697908	0,992961	
		NT	Si Low	Si High			NT	Si Low	Si High
PRO	NT		0,054497	0,317964	H2O2	NT		0,165002	0,090197
	Si Low	0,054497		0,598575		Si Low	0,165002		0,944077
	Si High	0,317964	0,598575			Si High	0,090197	0,944077	
		NT	Si Low	Si High			NT	Si Low	Si High
CAT	NT		0,90723	0,136602	APX	NT		0,581703	0,987861
	Si Low	0,90723		0,278558		Si Low	0,581703		0,492142
	Si High	0,136602	0,278558			Si High	0,987861	0,492142	
		NT	Si Low	Si High					
LOX	NT		0,878635	0,839059					

	Si Low	0,878635		0,996429
	Si High	0,839059	0,996429	

Table S7. Gladis, Tukey HSD Multiple Comparison for plants parts. L= leaf; R= root; F= fruit.

		L	R	F			L	R	F
**TP**	**L**		5,13E-09	0,721169	**MDA**	**L**		1,99E-05	0,001662
	**R**	5,13E-09		5,13E-09		**R**	1,99E-05		7,92E-09
	**F**	0,721169	5,13E-09			**F**	0,001662	7,92E-09	
		**L**	**R**	**F**			**L**	**R**	**F**
**SOD**	**L**		0,003263	0,945274	**POD**	**L**		0,077018	0,951545
	**R**	0,003263		0,001479		**R**	0,077018		0,137172
	**F**	0,945274	0,001479			**F**	0,951545	0,137172	
		**L**	**R**	**F**			**L**	**R**	**F**
**GST**	**L**		9,12E-06	0,746472	**GPX**	**L**		5,26E-09	0,00761
	**R**	9,12E-06		1,57E-06		**R**	5,26E-09		1,05E-07
	**F**	0,746472	1,57E-06			**F**	0,00761	1,05E-07	
		**L**	**R**	**F**			**L**	**R**	**F**
**PRO**	**L**		0,99904	0,011343	**H2O2**	**L**		3,12E-05	0,00519
	**R**	0,99904		0,012507		**R**	3,12E-05		0,122759
	**F**	0,011343	0,012507			**F**	0,00519	0,122759	
		**L**	**R**	**F**			**L**	**R**	**F**
**CAT**	**L**		0,000158	0,279916	**APX**	**L**		7,14E-08	0,666382
	**R**	0,000158		3,31E-06		**R**	7,14E-08		1,56E-08
	**F**	0,279916	3,31E-06			**F**	0,666382	1,56E-08	
		**L**	**R**	**F**					
**LOX**	**L**		0,998458	0,212721					
	**R**	0,998458		0,194595					
	**F**	0,212721	0,194595						

Table S8. Aragon, Tukey HSD Multiple Comparison per treatments. NT= not treated; Si Low= low Si treatment; Si High= High Si treatment.

		NT	Si Low	Si High			NT	Si Low	Si High
**Fe**	**NT**		0,975506	0,871241	**Al**	**NT**		0,072962	0,994346
	**Si Low**	0,975506		0,757783		**Si Low**	0,072962		0,059301
	**Si High**	0,757783	0,757783			**Si High**	0,994346	0,059301	
		**NT**	**Si Low**	**Si High**			**NT**	**Si Low**	**Si High**
**Cu**	**NT**		0,357949	0,98475	**Mn**	**NT**		0,826653	0,907161
	**Si Low**	0,357949		0,279957		**Si Low**	0,826653		0,984414
	**Si High**	0,98475	0,279957			**Si High**	0,907161	0,984414	
		**NT**	**Si Low**	**Si High**			**NT**	**Si Low**	**Si High**
**Na**	**NT**		0,685842	0,478433	**Ni**	**NT**		0,59931	0,876226
	**Si Low**	0,685842		0,937775		**Si Low**	0,59931		0,879511
	**Si High**	0,478433	0,937775			**Si High**	0,876226	0,879511	
		**NT**	**Si Low**	**Si High**			**NT**	**Si Low**	**Si High**
**Zn**	**NT**		0,199143	0,089437	**Mo**	**NT**		0,966574	0,751348
	**Si Low**	0,199143		0,900714		**Si Low**	0,966574		0,600343
	**Si High**	0,089437	0,900714			**Si High**	0,751348	0,600343	
		**NT**	**Si Low**	**Si High**			**NT**	**Si Low**	**Si High**
**Ca**	**NT**		0,600116	0,696357	**K**	**NT**		0,904739	0,930817
	**Si Low**	0,600116		0,986548		**Si Low**	0,904739		0,997609
	**Si High**	0,696357	0,986548			**Si High**	0,930817	0,997609	
**P**		**NT**	**Si Low**	**Si High**	**Mg**			**Si Low**	**Si High**

	**NT**		0,492274	0,525767		**NT**		0,987975	0,955043
	**Si Low**	0,492274		0,998243		**Si Low**	0,987975		0,90038
	**Si High**	0,525767	0,998243			**Si High**	0,955043	0,90038	
**Si**		**NT**	**Si Low**	**Si High**					
	**NT**		0,742194	0,410401					
	**Si Low**	0,742194		0,124872					
	**Si High**	0,410401	0,124872						

Table S9. Aragon, Tukey SHD Multiple Comparison per parts of the Plant. L= leaf; R= root; F= fruit.

		L	R	F			L	R	F
**Fe**	**L**		0,005457	1,01E-05	**Al**	**L**		0,696784	0,648009
	**R**	0,005457		0,047151		**R**	0,696784		0,996503
	**F**	1,01E-05	0,047151			**F**	0,648009	0,996503	
**Cu**		**L**	**R**	**F**	**Mn**		**L**	**R**	**F**
	**L**		0,690187	0,399548		**L**		7,75E-05	5,13E-09
	**R**	0,690187		0,876568		**R**	7,75E-05		5,13E-09
	**F**	0,399548	0,876568			**F**	5,13E-09	5,13E-09	
**Na**		**L**	**R**	**F**	**Ni**		**L**	**R**	**F**
	**L**		0,581967	5,19E-09		**L**		0,094141	0,026472
	**R**	0,581967		5,54E-09		**R**	0,094141		0,818949
	**F**	5,19E-09	5,54E-09			**F**	0,026472	0,818949	
**Zn**		**L**	**R**	**F**	**Mo**		**L**	**R**	**F**
	**L**		0,960787	0,141613		**L**		0,015497	0,028065
	**R**	0,960787		0,225889		**R**	0,015497		0,962826
	**F**	0,141613	0,225889			**F**	0,028065	0,962826	
**Ca**		**L**	**R**	**F**	**K**		**L**	**R**	**F**
	**L**		0,22904	0,001167		**L**		0,028093	0,001521
	**R**	0,22904		0,060907		**R**	0,028093		0,45099
	**F**	0,001167	0,060907			**F**	0,001521	0,45099	
**P**		**L**	**R**	**F**	**Mg**		**L**	**R**	**F**
	**L**		0,587627	0,005133		**L**		0,000217	1E-07
	**R**	0,587627		0,049809		**R**	0,000217		0,009729
	**F**	0,005133	0,049809			**F**	1E-07	0,009729	
**Si**		**L**	**R**	**F**					
	**L**		0,000116	0,739361					
	**R**	0,000116		0,000758					
	**F**	0,739361	0,000758						

Table S10. Gladis, Tukey HSD Multiple Comparison per treatments. NT= not treated; Si Low= low Si treatment; Si High= High Si treatment.

		NT	Si Low	Si High			NT	Si Low	Si High
**Fe**	**NT**		0,576927	0,446876	**Al**	**NT**		0,558452	0,500458
	**Si Low**	0,576927		0,973764		**Si Low**	0,558452		0,994862
	**Si High**	0,446876	0,973764			**Si High**	0,500458	0,994862	
**Cu**		**NT**	**Si Low**	**Si High**	**Mn**		**NT**	**Si Low**	**Si High**
	**NT**		0,913224	0,696143		**NT**		0,244087	0,477855
	**Si Low**	0,913224		0,451021		**Si Low**	0,244087		0,883724
	**Si High**	0,696143	0,451021			**Si High**	0,477855	0,883724	
**Na**		**NT**	**Si Low**	**Si High**	**Ni**		**NT**	**Si Low**	**Si High**
	**NT**		0,034238	0,053902		**NT**		0,13959	0,053526
	**Si Low**	0,034238		0,975488		**Si Low**	0,13959		0,877828
	**Si High**	0,053902	0,975488			**Si High**	0,053526	0,877828	

Zn		NT	Si Low	Si High
	NT		0,136061	0,687561
	Si Low	0,136061		0,487907
	Si High	0,687561	0,487907	

Mo		NT	Si Low	Si High
	NT		0,269413	0,937359
	Si Low	0,269413		0,151376
	Si High	0,937359	0,151376	

Ca		NT	Si Low	Si High
	NT		0,593613	0,581334
	Si Low	0,593613		0,999779
	Si High	0,581334	0,999779	

K		NT	Si Low	Si High
	NT		0,188265	0,990438
	Si Low	0,188265		0,234672
	Si High	0,990438	0,234672	

P		NT	Si Low	Si High
	NT		0,685173	0,386772
	Si Low	0,685173		0,095787
	Si High	0,386772	0,095787	

Mg		NT	Si Low	Si High
	NT		0,301316	0,994821
	Si Low	0,301316		0,347218
	Si High	0,994821	0,347218	

Si		NT	Si Low	Si High
	NT		0,859037	0,910719
	Si Low	0,859037		0,992855
	Si High	0,910719	0,992855	

Table S11. Gladis, Tukey HSD Multiple Comparisons per parts of the plant. L= leaf; R= root; F= fruit.

Fe		L	R	F
	L		0,002762	0,221906
	R	0,002762		0,125195
	F	0,221906	0,125195	

Al		L	R	F
	L		0,411761	0,998672
	R	0,411761		0,438811
	F	0,998672	0,438811	

Cu		L	R	F
	L		6,65E-08	0,191698
	R	6,65E-08		3,84E-06
	F	0,191698	3,84E-06	

Mn		L	R	F
	L		0,04423	6,84E-07
	R	0,04423		0,000356
	F	6,84E-07	0,000356	

Na		L	R	F
	L		0,044097	0,0008
	R	0,044097		0,230158
	F	0,0008	0,230158	

Ni		L	R	F
	L		0,229901	0,055071
	R	0,229901		0,729248
	F	0,055071	0,729248	

Zn		L	R	F
	L		0,835571	0,000304
	R	0,835571		0,001284
	F	0,000304	0,001284	

Mo		L	R	F
	L		0,954583	0,911811
	R	0,954583		0,765345
	F	0,911811	0,765345	

Ca		L	R	F
	L		0,565244	1,7E-06
	R	0,565244		2,07E-05
	F	1,7E-06	2,07E-05	

K		L	R	F
	L		0,252514	0,050739
	R	0,252514		0,670294
	F	0,050739	0,670294	

P		L	R	F
	L		0,009261	0,269459
	R	0,009261		0,241966
	F	0,269459	0,241966	

Mg		L	R	F
	L		1,47E-06	6,16E-08
	R	1,47E-06		0,340045
	F	6,16E-08	0,340045	

Si		L	R	F
	L		5,13E-09	0,447138
	R	5,13E-09		5,13E-09
	F	0,447138	5,13E-09	

References

48. Ali, N.; Schwarzenberg, A.; Yvin, J.C.; Hosseini, S.A. Regulatory Role of Silicon in Mediating Differential Stress Tolerance Responses in Two Contrasting Tomato Genotypes under Osmotic Stress. *Front. Plant Sci.* **2018**, *9*, 1–16, doi:10.3389/fpls.2018.01475.

73. Zhang, Y.; Liang, Y.; Zhao, X.; Jin, X.; Hou, L.; Shi, Y.; Ahammed, G.J. Silicon Compensates Phosphorus Deficit-Induced Growth Inhibition by Improving Photosynthetic Capacity, Antioxidant Potential, and Nutrient Homeostasis in Tomato. Agronomy **2019**, 9, 1–16, doi:10.3390/agronomy9110733.
104. Al-aghabary, K.; Zhu, Z.; Shi, Q. Influence of Silicon Supply on Chlorophyll Content, Chlorophyll Fluorescence, and Antioxidative Enzyme Activities in Tomato Plants under Salt Stress. *J. Plant Nutr.* **2004**, *27*, 2101–2115, doi:10.1081/LPLA-200034641.
105. Cao, B.; Wang, L.; Gao, S.; Xia, J.; Xu, K. Silicon-Mediated Changes in Radial Hydraulic Conductivity and Cell Wall Stability Are Involved in Silicon-Induced Drought Resistance in Tomato. *Protoplasma* **2017**, *254*, 2295–2304, doi:10.1007/s00709-017-1115-y.
106. Cao, B.; Ma, Q.; Xu, K. Silicon Restrains Drought-Induced ROS Accumulation by Promoting Energy Dissipation in Leaves of Tomato. *Protoplasma* **2020**, *257*, 537–547, doi:10.1007/s00709-019-01449-0.
107. Ghareeb, H.; Bozsó, Z.; Ott, P.G.; Repenning, C.; Stahl, F.; Wydra, K. Transcriptome of Silicon-Induced Resistance against *Ralstonia Solanacearum* in the Silicon Non-Accumulator Tomato Implicates Priming Effect. *Physiol. Mol. Plant Pathol.* **2011**, *75*, 83–89, doi:10.1016/j.pmpp.2010.11.004.
108. Ghareeb, H.; Bozsó, Z.; Ott, P.G.; Wydra, K. Silicon and *Ralstonia Solanacearum* Modulate Expression Stability of Housekeeping Genes in Tomato. *Physiol. Mol. Plant Pathol.* **2011**, *75*, 176–179, doi:10.1016/j.pmpp.2011.02.003.
109. Gunes, A.; Inal, A.; Bagci, E.G.; Pilbeam, D.J. Silicon-Mediated Changes of Some Physiological and Enzymatic Parameters Symptomatic for Oxidative Stress in Spinach and Tomato Grown in Sodic-B Toxic Soil. *Plant Soil* **2007**, *290*, 103–114, doi:10.1007/s11104-006-9137-9.
110. Heine, G.; Tikum, G.; Horst, W.J. The Effect of Silicon on the Infection by and Spread of *Pythium Aphanidermatum* in Single Roots of Tomato and Bitter Gourd. *J. Exp. Bot.* **2007**, *58*, 569–577, doi:10.1093/jxb/erl232.
111. Huang, C.H.; Roberts, P.D.; Datnoff, L.E. Silicon Suppresses Fusarium Crown and Root Rot of Tomato. *J. Phytopathol.* **2011**, *159*, 546–554, doi:10.1111/j.1439-0434.2011.01803.x.
112. Khan, A.; Kamran, M.; Imran, M.; Al-Harrasi, A.; Al-Rawahi, A.; Al-Amri, I.; Lee, I.J.; Khan, A.L. Silicon and Salicylic Acid Confer High-PH Stress Tolerance in Tomato Seedlings. *Sci. Rep.* **2019**, *9*, 1–16, doi:10.1038/s41598-019-55651-4.
113. Khan, A.; Khan, A.L.; Imran, M.; Asaf, S.; Kim, Y.H.; Bilal, S.; Numan, M.; Al-Harrasi, A.; Al-Rawahi, A.; Lee, I.J. Silicon-Induced Thermotolerance in *Solanum Lycopersicum* L. via Activation of Antioxidant System, Heat Shock Proteins, and Endogenous Phytohormones. *BMC Plant Biol.* **2020**, *20*, 1–18, doi:10.1186/s12870-020-02456-7.
114. Li, H.; Zhu, Y.; Hu, Y.; Han, W.; Gong, H. Beneficial Effects of Silicon in Alleviating Salinity Stress of Tomato Seedlings Grown under Sand Culture. *Acta Physiol. Plant.* **2015**, *37*, doi:10.1007/s11738-015-1818-7.
115. Muneer, S.; Park, Y.G.; Manivannan, A.; Soundararajan, P.; Jeong, B.R. Physiological and Proteomic Analysis in Chloroplasts of *Solanum Lycopersicum* L. under Silicon Efficiency and Salinity Stress. *Int. J. Mol. Sci.* **2014**, *15*, 21803–21824, doi:10.3390/ijms151221803.
116. Muneer, S.; Jeong, B.R. Proteomic Analysis of Salt-Stress Responsive Proteins in Roots of Tomato (*Lycopersicon Esculentum* L.) Plants towards Silicon Efficiency. *Plant Growth Regul.* **2015**, *77*, 133–146, doi:10.1007/s10725-015-0045-y.
117. Romero-Aranda, M.R.; Jurado, O.; Cuartero, J. Silicon Alleviates the Deleterious Salt Effect on Tomato Plant Growth by Improving Plant Water Status. *J. Plant Physiol.* **2006**, *163*, 847–855, doi:10.1016/j.jplph.2005.05.010.
118. Shi, Y.; Zhang, Y.; Han, W.; Feng, R.; Hu, Y.; Guo, J.; Gong, H. Silicon Enhances Water Stress Tolerance by Improving Root Hydraulic Conductance in *Solanum Lycopersicum* L. *Front. Plant Sci.* **2016**, *7*, 1–15, doi:10.3389/fpls.2016.00196.
119. Wu, J.; Guo, J.; Hu, Y.; Gong, H. Distinct Physiological Responses of Tomato and Cucumber Plants in Silicon-Mediated Alleviation of Cadmium Stress. *Front. Plant Sci.* **2015**, *6*, 1–14, doi:10.3389/fpls.2015.00453.
120. Jacquemond, M.; Verdin, E.; Dalmon, A.; Guilbaud, L.; Gognalons, P. Serological and Molecular Detection of Tomato Chlorosis Virus and Tomato Infectious Chlorosis Virus in Tomato. *Plant Pathol.* **2009**, *58*, 210–220, doi:10.1111/j.1365-3059.2008.01959.x.
121. Mascia, T.; Santovito, E.; Gallitelli, D.; Cillo, F. Evaluation of Reference Genes for Quantitative Reverse-Transcription Polymerase Chain Reaction Normalization in Infected Tomato Plants. *Mol. Plant Pathol.* **2010**, *11*, 805–816, doi:10.1111/j.1364-3703.2010.00646.x.
122. Poór, P.; Takács, Z.; Patyi, G.; Borbély, P.; Bencsik, O.; Szekeres, A.; Tari, I. Dark-Induced Changes in the Activity and the Expression of Tomato Hexokinase Genes Depend on the Leaf Age. *South African J. Bot.* **2018**, *118*, 98–104, doi:10.1016/j.sajb.2018.06.006.
123. Guo, J.; Wang, M.H. Characterization of the Phenylalanine Ammonia-Lyase Gene (SlPAL5) from Tomato (Solanum Lycopersicum L.). *Mol. Biol. Rep.* **2009**, *36*, 1579–1585, doi:10.1007/s11033-008-9354-9.
124. Saand, M.A.; Xu, Y.P.; Li, W.; Wang, J.P.; Cai, X.Z. Cyclic Nucleotide Gated Channel Gene Family in Tomato: Genome-Wide Identification and Functional Analyses in Disease Resistance. *Front. Plant Sci.* **2015**, *6*, doi:10.3389/fpls.2015.00303.
125. Vatansever, R.; Ozyigit, I.I.; Filiz, E.; Gozukara, N. Genome-Wide Exploration of Silicon (Si) Transporter Genes, Lsi1 and Lsi2 in Plants; Insights into Si-Accumulation Status/Capacity of Plants. BioMetals. **2017**, 30, 1–16, doi:10.1007/s10534-017-9992-2.
